# National and ethnic human mutation database: A need of the day

**DOI:** 10.4103/0971-6866.44997

**Published:** 2008

**Authors:** A. C. Gorakshakar, K. Ghosh

**Affiliations:** National Institute of Immunohaematology, 13^th^ Floor, New Multistoried Bldg, K.E.M. Hospital Campus, Parel, Mumbai-400 012, India

Bioinformatics is a relatively new discipline. It is a field of science in which Computer science, Mathematics, Molecular biology and Information technology merges to form a single discipline. Database development, sequence alignment, protein structure prediction, RNA folding, evolutionary tree construction are some of the areas covered under this discipline.

A biological database is a large organized body of persistent data usually associated with computerised software. It can be considered as electronic filing cabinets which store large amount of data in a systematic manner. One can update, query or retrieve components of data stored within the system. In 1960, Margaret Dayhoff established the first database of protein sequence while in 1982 (five years after the DNA sequencing techniques were discovered), the first nucleotide sequence database, GenBank was established. Databases are classified as Universal (eg. OMIM, HGMD) and Locus or Disease specific. [e.g. CFTR (Cystic Fibrosis), vWF(von Willebrand Factor), HAEMA (Haemophilia A), G6PD] or Primary (nucleotide sequence) and secondary (Protein sequence) database. At present there are number of databases on specific genes and proteins pertaining to human, animals, plants, bacteria etc.

In 1967, Victor McKusick started collecting data on human mutations. This ultimately resulted into OMIM (Online Mendelian Inheritance in Man) which mainly gives the list of Mendelian monogeneic disorders. It gives a lot of information about mutations, however all entries are not fully comprehensive. Human Gene Mutation Database (HGMD) was established in April 1996 by David Cooper[1] at the University of Wales, College of Medicine, Cardiff. It provides up to date and comprehensive information about inherited human gene lesions. In the late 90's prominent human geneticists from all over the world proposed to form a Federation of Locus Specific Data Bases (LSDB) as the mode of collecting accurate lists of mutations from various populations. This ultimately led to the formation of Mutation Database Initiative (MDI) which subsequently became a society, known as Human Genome Variation Society (HGVS). This was the product of the European Consortium involving the Karolinska Institute (Sweden), the European Bioinformatics Institute (UK) and the European Molecular Biology Laboratory (Germany). Its initial activity was to develop the databases of mutations seen in different populations and countries. This was done by inviting and promoting summaries of mutations, which is called ‘Mutation Updates’ for publication in the ‘Human Mutation’ journal. This helped to develop mutation databases of different countries and populations.[2–4] Recent databases also reports on phenotypic consequences, mode of inheritance and non disease related polymorphisms.[5]

Today novel DNA sequencing and genotyping techniques are being used to detect mutations. Such mutations are being identified at an exponential rate by researchers in various population groups of different countries. Currently publishing only mutations in a particular gene is difficult because many journals do not accept such articles. Similarly many laboratories do not consider reporting of such data as an essential activity. In such circumstances incorporating the mutation data in the appropriate database is the best way of reporting the DNA variation and eventually this could replace the conventional scientific publication as the predominant form of communication.

Regional or Ethnic Databases provide valuable data for the study of population history, genetic testing and disease association studies. Spectrum of mutations in a particular ethnic group helps to devise a strategy to detect mutations or to run prenatal diagnosis programs. It also enhances awareness about the various genetic disorders and provides insights into the genographic history of human populations.

There are very few reports from India which provide regional and ethnic data on the spectrum of genetic disorders. A search on the OMIM using the term ‘Indian sub-continent, gives 14 entries while a separate search for India yields 72 entries.[6] This resource is extremely important. However systematic cataloguing of molecular lesions of these traits is not done. Certain disorders like thalassaemia or hemophilia show a wide spectrum of mutations. Hence cataloguing this data in the form of database would help us to assess their overall burden and spectrum in various states/ communities in India.

On the other hand countries like Iran, Singapore,[7] Turkistan have their regional human mutation databases. In this issue we are happy to see that an attempt has been made by Ilham Ratbi from Morocco to develop ‘The Moroccan Human Mutation Database (MoHuMuDa)’.192 mutations and 123 diseases seen in Moroccan population are listed here. If other countries follow these steps; we hope that it would help diagnosticians, genetic counselors and population geneticists tremendously.
